# Identifying untapped legal capacity to promote multi-level and cross-sectoral coordination of natural resource governance

**DOI:** 10.1007/s11625-023-01424-y

**Published:** 2023-11-08

**Authors:** Nicola Harvey, Ahjond Garmestani, Craig R. Allen, Anoeska Buijze, Marleen van Rijswick

**Affiliations:** 1 Utrecht University Centre for Water, Oceans and Sustainability Law, Utrecht University, 3584 BH Utrecht, The Netherlands; 2 U.S. Environmental Protection Agency, 1 Sabine Island Drive, Gulf Breeze, FL, USA; 3 Center for Resilience in Agricultural Working Landscapes, University of Nebraska-Lincoln, Lincoln, NE, USA; 4 Department of Environmental Sciences, Emory University, Atlanta, GA, USA

**Keywords:** Administrative law, Climate change, Coordination, Environmental law, Natural resource governance, Water

## Abstract

Natural resource governance in the face of climate change represents one of the seminal challenges of the Anthropocene. A number of innovative approaches have been developed in, among others, the fields of ecology, governance, and sustainability sciences for managing uncertainty and scarcity through a coordinated approach to natural resource governance. However, the absence of an enabling legal and regulatory framework has been identified in the literature as one of the primary barriers constraining the formal operationalization of these governance approaches. In this paper, we show how these approaches provide tools for analyzing procedural mandates across governmental levels and sectors in the natural resource governance space. We also find that there has been inadequate consideration of the potential in existing laws and regulations for cross-sectoral and multi-level coordination of natural resource governance. On this basis, we develop and apply a protocol that draws on the traditional legal method of doctrinal analysis to demonstrate how to identify existing, untapped legal capacity to promote coordinated governance of natural resources through an in-depth case study of water resources in South Africa. We then show how these untapped capacities within existing legal structures may be operationalized to improve natural resource governance. Further, this protocol is portable to other countries, provinces (states), and localities around the world.

## Introduction

Natural resource governance is embedded in laws and institutions. Administrative (and in some jurisdictions constitutional) law empowers public officials to act, to decide, and formulate law and policy on matters pertaining to the governance of natural resources within clearly delineated areas of competency. When considering governance within a single resource sector, for example the water sector, sectoral law will further regulate the management of the particular resource in question. The sectoral legal framework should, however, always be understood in relation to the broader administrative legal regime: using our case study to demonstrate this point, the management and provision of water resources in South Africa is regulated by an extensive body of general environmental legislation (for example, the National Environmental Management Act) as well as sectoral legislation (for example, South Africa’s National Water Act) which, when contravened, can be enforced within a court of law. Understanding the role of law in shaping natural resource governance is thus essential, particularly when investigating or developing new governance arrangements to improve the management of natural resources.

Legal systems are intentionally constructed around a preference for maintaining the status quo through the promotion of stability, certainty, and predictability (Cosens et al. 2017; Ruhl 2012). The process of change is thus purposely slowed via checks and balances among the branches of government such that multi-stakeholder deliberation is fostered (Cosens et al. 2017). This prioritization of stabilization in the legal system may result in a mismatch with the dynamic nature of the natural resources that it regulates (Allen et al. 2011). This is because natural resource governance is characterized by ‘(1) high degrees of uncertainty; (2) complexity resulting from multiple variables and nonlinear interactions; (3) interconnectedness—among issues, across landscapes, and between people and place; and (4) persistent, possibly dramatic, change’ (Scarlett 2013). The varying levels of uncertainty and unpredictability characteristic of the natural resource governance context do not mesh well with legal certainty (Allen et al. 2011; Garmestani and Benson 2013). As such, decision-making in the context of natural resource governance presents information challenges, communication challenges, and action challenges (Scarlett 2013). This has prompted calls for institutional and organizational flexibility such that learning may be incorporated into natural resource governance which necessitates coordination and collaboration in policy and decision-making. In response to this need, a number of innovative approaches have been developed that strive to manage scarcity and complexity across natural resource systems through the promotion of inter-sectoral and/or cross-scale coordination and collaboration, including adaptations of panarchy theory to natural resource governance (Allen et al. 2014; Garmestani and Benson 2013; Gunderson and Holling 2002), adaptive management and governance (Allen and Gunderson 2011; Folke et al. 2005; Walker et al. 2004), the water, energy, and food nexus (Endo and Oh 2018; Biggs et al. 2015; Hoff 2011), and reflexive governance (Voß and Bornemann 2011; Voß et al. 2006; Dedeurwaerdere 2005; Lenschow 2002).

If, in pursuing the goal of stability, the rigidity of the law frustrates a coordinated approach to natural resource governance as is required to effectively respond to the dynamic nature of water, then we must conclude that the law itself requires amendment. However, a call for legal amendment premised upon only an assumption of rigidity derived from recognition of the law’s goal of stability but absent of a creative and thorough interpretation of existing legislation, is to oversimplify the issue. The law should first be thoroughly assessed for untapped capacity to promote a coordinated approach to natural resource governance (Garmestani et al. 2019), which need not inherently conflict with the normative goal of promoting stability. Should such untapped capacity exist, it may then be utilized, thereby avoiding the need for large-scale legal reform. This is desirable given that legal reform requires a lengthy procedural process often thwarted by layered bureaucracy and insufficient political will and is thus not likely to happen as swiftly as impending environmental issues require (Craig et al. 2020).

Through a case study analysis of the general and sectoral law regulating water resources in South Africa, we answer the following research question:

What untapped capacity exists within the existing formal water law framework in South Africa to promote greater degrees of coordination across different levels of government (national, provincial and local levels) and across different governance sectors (i.e., can water officials coordinate with officials in other governmental departments such as with energy or health officials)?

In answering this research question we develop a protocol that aids in identifying existing, untapped legal capacity to promote coordinated governance of water resources in South Africa. Notwithstanding the jurisdictional specificity and sectoral focus, the use of the protocol and structure of our analysis serves as a model for the evaluation of existing laws for untapped capacity to promote coordination of different governance regimes around the world.

Importantly, the focus of this research on the formal institutional structure and its underlying regulatory framework should not be interpreted to diminish informal means of coordination. In fact, informal interpersonal interaction between public actors within and across governmental departments represents an important means of promoting coordination in natural resource governance, as does local level community-based arrangements (Oldfield and Greyling 2015; Chaffin et al. 2016; Enqvist et al. 2020; Clement 2022). It is the aim of this research to complement existing accounts of informal means of coordination with an understanding of the correlating role of the formal regulatory framework (i.e., laws and institutions) in shaping coordination practices. It is quite clear that this latter perspective has been understudied and accounted for in research on natural resource governance. Here, we seek to advance research on the essential role of law and formal regulatory frameworks on natural resource governance with a case study of the water resources of South Africa.

## Background and context

Research on different forms of governance, such as research on adaptations of panarchy theory to environmental governance (Allen et al. 2014; Garmestani and Benson 2013; Gunderson and Holling 2002), adaptive management and governance (Allen and Gunderson 2011; Folke et al. 2005; Walker et al. 2004), the water, energy and food nexus (Endo and Oh 2018; Biggs et al. 2015; Hoff 2011), and reflexive governance (Voß and Bornemann 2011; Voß et al. 2006; Dedeurwaerdere 2005; Lenschow 2002) has attempted to address coordination across sectors and levels of government, aspiring for improved management of natural resources. The call for intergovernmental coordination in response to the demonstrated interdependence of natural resource systems and governance is a principal point of intersection of this scholarship (Folke et al. 2005). These studies establish a means of examining how different actors operating across sectors at different levels of government achieve their objectives in the natural resource governance space (Garmestani and Benson 2013).

In this section, we show how this literature provides tools for analyzing procedural mandates across governmental levels and sectors in the natural resource governance space and demonstrate a tendency to focus on coordination challenges. At the same time, we argue that the literature inadequately considers the existing potential for multi-level and cross-sectoral coordination in existing laws and regulations and as a result does not adequately consider the practical application of the coordination solutions it puts forward. We begin by briefly defining what we mean by ‘natural resource governance’, before summarizing the scholarly debates on coordination from different approaches to natural resource governance popular in contemporary literature. In this way a conceptual basis is established upon which the doctrinal analysis (standard legal analysis) may be conducted demonstrating how untapped capacity to coordinate within existing regulatory frameworks may be identified.

### Intergovernmental coordination in natural resource governance

Governance, for the purpose of this paper, refers to ‘the means through which collective goals are chosen, decisions are made, and action is taken to achieve the chosen goals’ (Cosens and Gunderson 2018, p. 5). The term governance is broader than government including within its scope the relationship between government and society and thus also the ways through which private actors, markets, and even interest-based networks self-organize to both mediate their own behavior and influence policy decisions (Folke et al. 2005; Huitema et al. 2009; Lemos and Agrawal 2006). Natural resource governance is that subset of collective action including the norms, institutions, structures and processes mediating human interaction with natural resource systems and ‘determines how power and responsibilities over natural resources are exercised, how decisions are taken, and how citizens participate in and benefit from the management of natural resources’ (Springer et al. 2021). Water resource governance is then concerned specifically with the relationship between citizens and water resources.

Coordination of natural resource governance here refers to the fostering of interactions between agents that can produce wanted or better outcomes as determined by some standard (Vatn 2012). Many perspectives exist from which coordination may be approached. We focused on coordination as a process, and more specifically with the associated strategies and mechanisms that governments use to coordinate public organizations or programs concerned with natural resource governance. The scope of this research is thus restricted to identifying untapped capacity for coordination in formal mechanisms found in law and policy in a public sector interorganizational context. Coordination in a public sector interorganizational context is here understood as constituting ‘the instruments and mechanisms that aim to enhance the voluntary or forced alignment of tasks and efforts’ of organizations within the public sector within and between policies, implementation, or management (Bouckaert et al. 2010). Multi-level and cross-sectoral coordination has been identified as necessary elements in the operationalization of a number of innovative approaches that have been developed to manage scarcity and complexity across natural resource systems.

Panarchy theory was developed by Gunderson and Holling to understand how human and ecological systems function and interact across scales (Gunderson and Holling 2002). Allen et al. (2014) defines panarchy as ‘a conceptual model that describes the ways in which complex systems of people and nature are dynamically organized and structured across scales of space and time’. When applied in the context of natural resource governance, panarchy responds to the recognition that human and natural systems interact in ‘complex, nonlinear ways, with multiple avenues for feedback among systems’ (Cosens and Gunderson 2018, p. 2). Governance of natural resources thus needs to be flexible and adaptive. Legal scholars argue that to be effective, tools for flexible and adaptable management require embedding within systems of law and governance to address the intersection of the social–ecological systems being governed and the legal system (Garmestani et al. 2009; Ruhl 2012). This in turn requires high levels of information sharing, cooperation, and coordination across stakeholders to enable flexible and adaptive responses to changes in the natural systems they interact with or control (Cosens and Gunderson 2018; Allen et al. 2014; Ruhl 2012; Garmestani et al. 2009).

Adaptive management and governance has been put forward by scholars as a means of handling uncertainty in natural resource governance (Cosens and Gunderson 2018; Garmestani et al. 2009). It does so by purposely and explicitly increasing knowledge (through learning), thereby decreasing uncertainty to allow for effective management of natural resources (Holling 1973; Walters 1986). The central concept in adaptive management is ‘that policy choices should be treated as deliberate, large-scale experiments; hence, policy choice should be treated at least partly as a problem of scientific experimental design’ and nested in an adaptive governance framework (Allen and Gunderson 2011, p. 1379). The literature on adaptive management and governance (particularly the application to water resources) increasingly highlights the institutional nature of barriers to practical success, including the absence of enabling regulatory and policy environments (Akamani 2016; Allen and Gunderson 2011). In this regard, studies have revealed that the failure to implement adaptive management is a result of factors such as the absence of shared decision-making among diverse stakeholders (Gregory et al. 2006); the inability of overlapping management agencies to effectively communicate and agree on the distribution of responsibilities for implementing an adaptive management plan (Gregory et al. 2006), a belief within public agencies that single best policies lend credibility (Walters 1997); an absence of processes promoting shared understanding and shared decision-making across diverse departments and stakeholders (Gregory et al. 2006); and lack of funding for the increased monitoring required to properly compare the outcomes of alternative policies (Walters 2007).

The water, energy, and food nexus has developed as a concept describing the linkages across water, energy, and food systems (Lawford et al. 2013; Weitz et al. 2017). The WEF nexus thus acknowledges the existing sectoral interdependencies and the correlating need to make use of potential synergies and manage trade-offs. To adequately respond to cross-sectoral interactions such that synergies may be identified and trade-offs managed requires a coordinated approach by government actors possessing the requisite mandate and powers to respond (Harvey 2023). To this end, numerous studies on the WEF nexus have demonstrated how political silos give rise to communication and collaboration barriers (Bhaduri et al. 2015; Daher and Mohtar 2015; Kaddoura and El Khatib 2017; Liu et al. 2017; Scott et al. 2015; Simpson and Jewitt 2019), differing values, goals, priorities, and cultures between governing sectors (Covarrubias 2019; van Gevelt 2020), and conflicting sectoral decision-making processes and limited cross-sectoral communication (Daher and Mohtar 2019; Howarth and Monasterolo 2016; Liu et al. 2018; White et al. 2017). Such issues not only exist between sectors, but also across the various levels of government, where different interests, powers, and incentives frustrate organizational coordination (Bergendahl et al. 2018; Daher and Mohtar 2019). The literature further emphasizes the necessity of engagement with a broad range of stakeholders extending beyond public officials to address silos (Bhaduri et al. 2015; Laspidou et al. 2020; Mohtar and Daher 2016; Olawuyi 2020; van Gevelt 2020; White et al. 2017). Additionally, as is the case in the adaptive management and governance literature, nexus scholars call on policy makers to consider and agree on a clear financial plan to accompany proposed interventions so as to ensure capacity to implement nexus approaches (Daher et al. 2019). Finally, nexus literature emphasizes the need for policy to reflect nonlinearity of resources, and demands a degree of flexibility and adaptability that is not ordinarily characteristic of policy design (Bhaduri et al. 2015; Kurian 2017; Liu et al. 2018).

Reflexive governance, defined more generally, is ‘the ability of a structure, process, or set of ideas to reconfigure itself in response to reflection on its performance’ (Dryzek and Pickering 2017, p. 353). Reflexivity in the narrower context of natural resource governance is concerned with social–ecological systems (as opposed to a focus on human systems alone) and refers to the ability for public actors to recognize and interpret signals from the physical resource systems and to rethink and reshape core governance values and practices accordingly (Dryzek and Pickering 2017). A link can be drawn between theories of reflexive governance and the contemporary scholarly recognition of the increased relevance of networked coordination structures over more traditional/hierarchical modes of coordination (McNutt and Rayner 2018). As such, reflexive governance scholars argue that the formal regulatory framework should create an enabling environment for reflexive (networked) coordination in this regard (Schutter and Lenoble 2010). The concept of reflexivity has been applied across a broad range of environmental issue areas (Feindt and Weiland 2018) including collaborative water and flood risk governance (Mees et al. 2018; Westling et al. 2014); transboundary marine spatial planning (Boström et al. 2016; van Tatenhove 2017); and to various topics related to governance of the food system including food security (Sonnino et al. 2014), food chain performance (Kirwan et al. 2017), and sustainable transitions of food and agricultural systems (Feindt 2012; Marsden 2013). One of the core barriers to a reflexive approach to natural resource governance beyond entrenched politics and power struggles is the necessity of horizontal learning, which is frustrated by rigid adherence to polarized policy positions (Durnova 2018) and/or the perceived need to maintain close networks (Gottschick 2018; McNutt and Rayner 2018). Further barriers include ‘inequalities of information, power imbalances, [and] the lack of access to networks’ as well as a lack of information sharing across governance networks (Feindt and Weiland 2018).

It is thus clear that a principal point of intersection across a number of different approaches to natural resource governance is the need for intergovernmental coordination in response to demonstrated sectoral interdependence and the uncertain and dynamic nature of natural resource systems (Garmestani and Benson 2013). Additionally, in all instances, the absence of an enabling legal and policy environment was identified as one of the primary barriers to the successful implementation of all of these governance approaches in practice (Ruhl and Fischman 2010). What constitutes an ‘enabling legal and regulatory environment’ is often not set out within the literature; the law is simply treated as a problem that hinders sound governance, without an analysis of the legal system in question. Our review of the literature found a number of characteristics that scholars identified as desirable in any legal and regulatory framework promoting coordination which we summarize in Table 2. Furthermore, it is important to note that in every instance, the conclusion surrounding the inadequacy of existing regulatory frameworks is reached without a thorough legal review of the regulatory framework in question. This is unsurprising given the lack of legal perspectives within the literature on natural resource governance, and the consequent failure to connect law with approaches to natural resource governance (but see Garmestani and Benson 2013).

Although a number of social and economic challenges may prevent the practical operationalization of any one of these approaches to natural resource governance, we are here focused on addressing the institutional and legal challenge of an absent supporting regulatory framework. It is our proposition that in many cases, the legal and regulatory framework can in fact support any one of these new approaches to natural resource governance and therefore represents far less of a barrier than existing scholarship has stated. The hidden or untapped capacity of an existing regulatory framework is derived from the unique character of the law. As the highly acclaimed legal philosopher Ronald Dworkin famously stated:

‘law is a social phenomenon. But its complexity, function, and consequence all depend on one special feature of its structure. Legal practice, unlike many other social phenomena, is argumentative’

(Dworkin 1986, p. 13).

The untapped capacity of an existing regulatory framework thus rests within the ability to creatively interpret existing law such that it may be validly applied in pursuance of a different result (Garmestani et al. 2019). Therefore, should the social scientist wish to assess whether there exists a supporting regulatory and policy environment for a new approach to natural resource governance, they should not expect to find a single existing regulatory framework that perfectly reflects the governance structure they wish to follow and expressed in the same (scientific) language of their new governance approach. This is unlikely to exist.

However, through the application of the interpretive method of the legal discipline, existing law should be reviewed for procedural rules and legal mechanisms that, through creative interpretation, enables an application of the same legal rule in a new but legally valid context, thus having a different effect and yielding a different desirable outcome in practice (Hoecke 2011). This outcome is one that enables the operationalization of the new approach to natural resource governance, as demonstrated extensively in the discussion section of this paper. This is not a radical proposal. Doctrinal analysis (legal analysis) is, after all, at the core of legal practice, and more broadly, there is no viable sustainability pathway without consideration of and accounting for the law (Garmestani et al. 2019; Ruhl 2012).

Doctrinal analysis requires a uniquely legal manner of engaging in descriptive or exploratory analysis on the basis of the interpretive method (Hoecke 2011). Additionally, where the research question is evaluating a legal state of affairs or seeking solution to a legal problem, the evaluation is grounded not in a theoretical framework but in a normative one (Taekema 2018). Legal doctrine is thus multifaceted, being a predominantly ‘hermeneutic discipline, with also empirical, argumentative, logical and normative elements’ (Hoecke 2011, p. 157). Doctrinal legal research thus organizes legal texts (e.g., laws, regulations), conducts analysis from an interpretative, hermeneutical perspective, and develops coherent arguments on the law based on logical deduction, inference, and normative claims (van Boom et al. 2018).

The purpose of this research is therefore to demonstrate the utility of connecting natural resource governance with the law. This is achieved by focusing on a principal point of intersection (i.e., intergovernmental coordination) across a number of different approaches to natural resource governance to demonstrate how doctrinal analysis may reveal untapped capacities in the existing legal system to promote coordination of governance. This analysis can be easily replicated across multiple resource sectors, for example in the governance of energy, food, and mineral resources. However, for this work we confined our case study to a single jurisdiction, namely South Africa, and a single sector, namely the water sector. This is because the primary purpose of this study is to demonstrate the utility of this approach (proof of concept) rather than conduct a full-scale doctrinal analysis of the entire regulatory framework of a country.

### Case study

South Africa is predicted to experience the largest decline in precipitation in the sub-Saharan African region, with concurrent risks of severe drought (Serdeczny et al. 2017). Indeed, in 2017, Cape Town (South Africa’s southernmost and second largest city) became the first ever major city in the world to nearly run out of water entirely (Sousa et al. 2018; Millington and Scheba 2021). The issue of scarcity is made more complex by the prevailing social and economic inequality within South Africa inherited from the racist Apartheid regime. From 1948 to the early 1990s, the National Party (being the governing party during this time) pursued an official and formal policy of segregation involving legal, political, and economic discrimination against all non-White persons (Dubow 2014). Progress in reversing Apartheid’s entrenched system of institutional racism has been slow, but the country now ‘has one of the most progressive constitutions in the world, with a bill of rights that foregrounds expanded socioeconomic rights’ (Francis and Webster 2019, p.788). This is significant because post-Apartheid South Africa is a constitutional democracy, making the Constitution the highest law in the land.

South Africa’s Constitution establishes a three-tiered structure of government, allocates powers across the three tiers of government, defines the nature and scope of such powers, and designates the procedural requirements for law making and decision-making across all spheres (i.e., national, provincial, and local) of government. Power to make general law and policy largely rests with national government, while the responsibility for delivery of services is held by local governments. Each level of government contains a number of departments responsible for administration of a particular public matter. Siloed management of natural resources can occur when government departments do not coordinate their policy and decision-making with other affected departments. Given that the climate change-induced threat of water scarcity coupled with the complex social and economic context promoting unequal access to water resources requires a rapid and effective response from within a siloed institutional structure of government, a clear need for effective coordination of natural resource governance arises in South Africa.

## Methods

A single instrumental case study design was used given that our focus was on a single issue (coordination in a public sector interorganizational context) rather than the ‘case’ itself (Yin 2009). Data collection was conducted via document reviews. Given the elevated status of the documents, namely binding law and official policy that is the primary source establishing the formal institutional structure and powers to coordinated government, we determined that document review alone provided sufficient data to answer our research question. The case study selected was the South African water resource governance regime, including the broader environmental legislation applicable to water governance (for example, the National Environmental Management Act). To assess the capacity for coordination across different levels of government, we focused the case study analysis on South African national level law and policy, Western Cape provincial level law and policy, and City of Cape Town local level law and policy, respectively. All three levels of government have open access electronic databases containing regularly updated versions of all law and policy currently in force.

Overarching legislation and policy, particularly those regulating the exercise of public powers, were selected first. This cannot be done on the basis of a keyword search given the risk of excluding relevant documents that have the effect of shaping the exercise of public power without mentioning the specific key word. Consequently, the traditional legal research method of doctrinal review was thus adopted. As such, the legal expertise of the authors and their underlying understanding of the legal system studied as a whole inform which legislation must be included in the analysis. All legislation that is necessary in determining how public power is assigned within the institutional structure of the government is thus included. In South Africa, this is simple: because it is a constitutional democracy, the primary and most supreme law regulating public powers is the Constitution of the Republic of South Africa. We therefore include the Constitution in the analysis as well as all of the laws, policy, and strategy documents that the Constitution expressly requires be enacted to designate specific powers. A total of 11 general documents were reviewed: laws (*n* = *8*) and policy (*n* = *3*).

To identify water sector legislation, a standard keyword search is possible. In South Africa, the provincial government does not have competence (i.e., power to make laws) over matters concerning water. Thus, documents from the national^1^ and local^2^ government’s respective electronic databases (document centers) were collected for review. The key word “water” was inputted into the electronic databases and results were filtered to display all law (i.e., acts or byelaws), policy, and official governmental strategy documents currently active (i.e., not repealed or replaced) in South Africa.

The generated list of documents was manually checked and documents were selected for review if they met 3 interrelated criteria: (1) addressed the management of water either in and of itself or in relation to another sector; (2) addressed or designated decision-making and/or planning powers regarding water resources; (3) technical regulations containing only standards for waste disposal or technical standards regarding water storage were excluded for irrelevance as they do not regulate intergovernmental powers or interactions. A total of 36 sectoral documents were included: laws (*n* = *30*), policy (*n* = *4*) and binding strategy or guidelines (*n* = *2*). All 47 documents (11 general and 36 sectoral) are presented in Table 1.

To analyze the selected documents, we developed a protocol drawing from the existing state of the art as analyzed in the preceding section in this paper, as well as from the research results of a number of more extensive literature reviews (Harvey 2023; Cosens and Gunderson 2018; Endo et al. 2017; Cosens et al. 2017; Voß and Bornemann 2011) in which the characteristics of a regulatory framework with capacity to promote interorganizational coordination were identified. This protocol is presented in Table 2. The selected documents were reviewed in detail and any provisions that reflected one or more characteristics were recorded in an Excel spreadsheet. The authors then had an overview of all the formal mechanisms for promoting coordination, and could apply deductive legal reasoning—as is standard within the legal method of traditional doctrinal review (Taekema 2018; Hutchinson 2015; Hutchinson and Duncan 2012)—to describe in what way such legal and policy mechanisms may be utilized to promote coordination in practice. This is described in the analysis below.

## Results

The results of the doctrinal review are summarized in Table 3 and set out in detail below.

### Capacity for coordination within the institutional structure of government

**Fig. 1:**
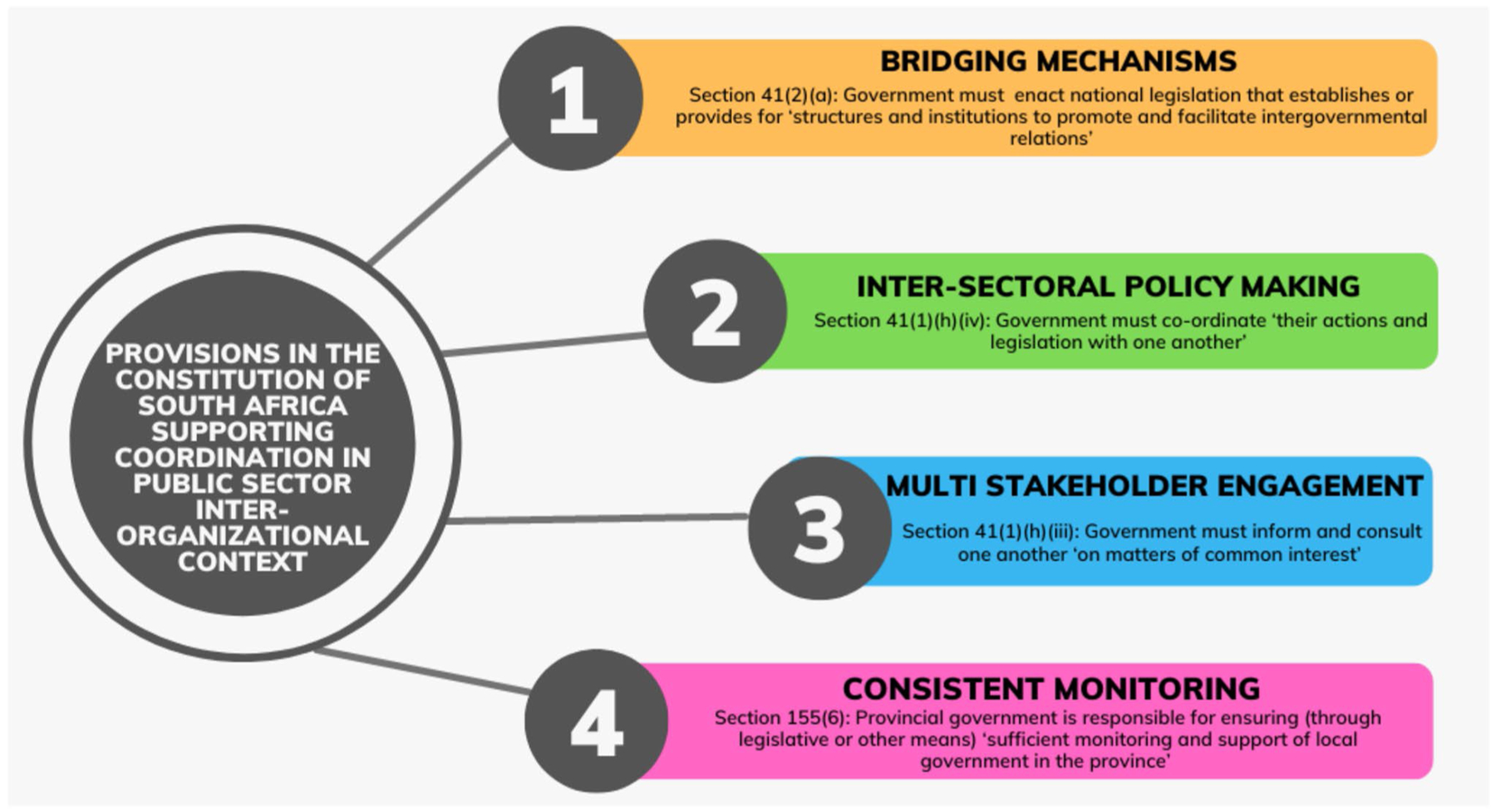
Summary of legal mechanisms in the Constitution of South Africa found to have capacity to promote coordination of governance

We reviewed every clause of South Africa’s Constitution to identify mechanisms or procedures reflecting one or more of the characteristics detailed in our protocol (Fig. 1, Table 2).

Section 41 of the Constitution contains the Principles of Cooperative Governance, the underlying purpose of which is to limit interdepartmental conflict and competition across all levels and sectors of government. The cooperative government principles have been interpreted by the South African Constitutional Court (the highest court in the land) as constituting an ‘express provision that all spheres of government must exercise their powers and functions in a manner that does not encroach on the geographical, functional or institutional integrity of government in another sphere’ (Certification of the Constitution of the Republic of South Africa 1996). Given that Section 41(1)(h) of the Constitution requires that government officials across departments coordinate and consult with one another, and further that coordination and consultation have been viewed as the two essential facets of collaboration (Gulati et al. 2012), it is unsurprising that three of the principles contain characteristics that we have identified as promoting coordination.

In practical terms, the effect of these provisions can be significant. Given that the Constitution is the highest law in the land, it is required that (1) all law and policy currently in force be compliant with the Constitution and be interpreted in light of its provisions; and (2) that all action taken by public actors must be in compliance with the Constitution. For the purposes of the present research question, this means that the various governmental departments responsible for the management of water (and in fact for the management of any environmental matter) must legislate, create policy, and perform their functions in a coordinated manner with other governmental departments and with the inclusion of relevant consultations. To fail to do so would be in breach of the Constitution. In this case, untapped capacity for coordination exists because the Constitutional Court in *National Gambling Board v Premier KwaZulu-Natal and Others* (CCT32/01) and in *Western Cape Minister of Education and Others v Governing Body of Mikro Primary School and Another* (SCA140/05) confirmed the justiciability of section 41. This means that if a law/policy contravenes (does not comply with) the section 41 requirement to coordinate, consult, and cooperate in the formulation of such law/policy, or if the law/policy unjustifiably conflicts with the law/policy of another government department, that law or policy may be taken on review. Should the Constitutional Court determine that the law/policy contravenes section 41, it can be declared invalid. The very limited number of cases invoking section 41 demonstrates a lack of reliance on this section (Gamper 2010). So arises the untapped capacity within the law to compel coordination and cooperation in the law/ policy making process. Thus, we can already begin to see a basis for the promotion of interorganizational coordination within the institutional structure of government.

### Capacity for coordination within the overarching legislative and policy framework

Our review of the overarching legislative and policy framework against our coordination protocol found that mechanisms reflecting all nine characteristics promoting coordination were present. The specific sections containing such mechanisms are too numerous to reproduce in full here. Thus, we grouped together those mechanisms from the different legal and policy documents that contained the same characteristics and formulated ten categories of coordinating mechanisms. Table 4 presents these ten categories alongside the correlating characteristic as identified in our coordination protocol. The third column of Table 4 gives examples of each category of mechanisms extracted from the document review. It is important to note that, given that the overarching documents are those regulating public power and public action, these mechanisms are generally applicable (i.e., they apply to all actions and decisions taken by government) and thus promote coordination not only within the water sector, but also across all resource sectors and levels of government.

Participation procedures and guiding principles are perhaps less widely recognized mechanisms for promoting coordination across governmental departments and their inclusion as categories of coordinating mechanisms in Table 3 requires brief elaboration.

Public participation requires engagement with all interested or affected stakeholders, as public officials from another department are empowered to participate in participatory proceedings where their interests are impacted. For example, “Background and context”(4)(f) of the National Environmental Management Act 107 of 1998 requires that participation of ‘all interested and affected parties in environmental governance’ be promoted. The official guidelines for the interpretation of this Act state that interested and affected parties include ‘any organ of state that may have jurisdiction over any aspect of the activity’ and thus allow for coordination between governmental departments through participation proceedings.

Guiding principles, though not directly promoting coordination, contribute to solving a core complexity in navigating natural resource governance when operationalizing coordinated approaches. A governance arrangement requiring closer coordination whether across sectors or levels of government will, at one point or another, necessitate a choice by public officials of which (sectoral) interests to prioritize and to what degree. As van Gevelt 2020 shows, these decisions are inherently subjective, shaped by ‘the values and objectives of stakeholders and decision-makers, procedural considerations and power relations between stakeholders’. Navigating inter-sectoral trade-offs is thus a complex matter. However, South African law’s explicit prioritization of certain value-laden principles within binding legislation aids public officials and stakeholders in their choice over which interests to prioritize.

Returning to the question of untapped capacity in the law, all provisions (sections of the law) set out in Table 4 are binding law. This means that failure to comply provides grounds to take the relevant government department to court to compel compliance via a court order. This is of course a formal and potentially time-consuming process, but often the mere threat of such a process is sufficient to compel compliance. Additionally, the existence of these legal provisions challenges the claims in the literature that existing legal and regulatory frameworks do not adequately support coordination across sectors and levels of government.

### Capacity for coordination within the sectoral legislative and policy framework

The majority of mechanisms promoting coordination identified within the overarching legislative and policy framework are also reflected within sectoral legislation (with categories 4 and 7 being the only exception). An eleventh category of mechanisms not found in the overarching legislation was identified in the sectoral legislation. The results are presented in Table 5.

As was the case in the overarching legislation, the capacity to coordinate here lies in the legal basis that these provisions provide for compelling compliance therewith. The claim that a key issue with operationalization of a new approach to natural resource governance rests in the absence of a legal framework adequately supporting such an approach is again challenged by the findings in Table 5. For example, the claim by administrative governance scholars (Gregory et al. 2006) and WEF nexus scholars (Bhaduri et al. 2015; Laspidou et al. 2020; Mohtar and Daher 2016; Olawuyi 2020; van Gevelt 2020; White et al. 2017) that there is an absence of legal processes promoting shared understanding and shared decision-making across diverse departments and stakeholders can be contrasted with section 10(2) of the National Water Act which states that ‘[i]n developing a catchment management strategy, a catchment management agency must consult with…(b) any organ of State which has an interest in the content, effect or implementation of the catchment management strategy.’ This notwithstanding, the presence of enabling legal mechanisms may not be sufficient to resolve coordination challenges. The practical implications of this are discussed in the section that follows.

## Discussion

Our analysis of the formal (legal and institutional) capacities in the South African legal system to coordinate water resource governance (natural resource governance), revealed evidence of a variety of mechanisms and processes promoting coordination across sectors and different levels of government. While each mechanism has particular virtues, none in isolation are a panacea (Peters 2017). Additionally, such mechanisms enable different forms of coordination of water resource governance: whereas some mechanisms depend upon the imposition of top-down forms of coordination, others serve to encourage coordination by means of individual interactions and bargaining among relevant public actors.

For example, within the overarching natural resource governance regime the National Environmental Management Act’s mandate for the development of environmental implementation plans (EIP’s) or environmental management plans (EMP’s) by national departments, as analyzed in “Capacity for coordination within the overarching legislative and policy framework”, represents a mechanism promoting top-down coordination of natural resources including water resources. This is because EIP’s and EMP’s ensure departments at the national level take steps to coordinate any environmental policies, plans and decisions with that of other national level departments. Given the hierarchical nature of law and policy, with legal instruments higher in the hierarchy generally being superior in effect (Garmestani et al. 2019), this will in turn impact the boundaries of action and decision-making for natural resources (again, including water resources) at provincial and local levels.

More specifically to water resource governance, section 1.4 of the binding National Water Resource Strategy of 2004 requires that catchment management agencies (being regional public bodies responsible for the management of water resources within their respective catchment areas) ensure that their water-related plans and programs are consonant with the plans and programs ‘of all other role players in the catchments they manage’. Section 1.4 thus goes on to require that catchment management agencies ‘establish co-operative relationships with a range of stakeholders, including other water management institutions, water services institutions, provincial and local government authorities, communities, water users ranging from large industries to individual irrigators, and other interested parties.’ In this way coordination of water resources is encouraged by means of individual interactions and bargaining among relevant public actors. Should public actors fail to meet this obligation the decision or action taken may be invalidated on the basis of failure to comply with section 1.4 of the National Water Resource Strategy of 2004, or in the alternative may be reviewed against section 41 of the Constitution and invalidated on the grounds of constitutional non-compliance. In both instances, the means of enforcement is legal action in court, the disadvantages of which we discuss later in this section.

The legal mechanisms identified not only enable different forms of coordination in water resource governance, but also envisage coordination at different stages of the decision-making process. This in turn shapes the nature and effect of the coordination achieved by the particular legal mechanism. By way of example, the implementation protocol envisaged by section 35 of the Intergovernmental Relations Framework Act in which organs of state define their respective roles and responsibilities ‘in implementing policy, exercising the statutory power, performing the statutory function or providing the [public] service’ provides for coordination at the stage of implementation of policy. However, section 24(1) of the Municipal Systems Act requires that planning undertaken by a municipality be aligned with and complement ‘the development plans and strategies of other affected municipalities and other organs of state’ and thus promotes coordination of water resource governance at the policy formulation stage.

Cognizance of the different forms of coordination is important when pursuing a governance approach that promotes coordination across sectors and scales. This is not, in principle, problematic. However, where coordinated governance of water resources is required to achieve specific aims, then voluntary mechanisms may be insufficient to ensure that such coordination does actually occur in practice. This is particularly the case when taking into consideration the role that power and self-interest plays in shaping decision-making by public actors (Scharpf 1994).

Broadly speaking, and with ramifications beyond South Africa, even under perfect conditions in which public actors make decisions solely based upon the pursuance of the public best interest, they may be unaware of the existing formal coordination mechanisms within the water resources regime that are available to them. In such instances two possible solutions arise: a clear mandate requiring and framework enabling coordination is necessary with a clear stipulation of the aim it is pursuing and at what scale and stage of decision-making coordination must take place. Alternatively, the legal framework can empower public actors affected by a decision in a particular department to notify such department of the need to coordinate, placing the voluntary coordination mechanism at the liberty of the party affected rather than the party making the decision. Absent of these alternatives, coordinated governance of water resources is less likely to occur unless such coordination is to the benefit of all parties involved.

In some contexts, it may be desirable to ensure the availability of a variety of formal coordinating mechanisms, but to allow public officials to make use of such mechanisms when it suits them. In this way space is created within the legal regime for public officials to develop creative solutions to particular water resource governance challenges at multiple scales (Garmestani and Benson 2013). For this approach, the availability of legal mechanisms absent of a specified mandate stipulating at what stage and in what manner such mechanisms must be used allows for varying instruments, or combinations of instruments, to be utilized to improve water resource governance by focusing upon processes (learning) instead of fixed or static predetermined aims.

Such flexibility and space for creativity in decision-making is particularly important in light of the fact that climate change, and indeed global change more generally, reflects increasing non-stationarity (Craig 2010). Non-stationarity in the face of very stationary or static governance structures is likely to erode resilience over time. When resilience is exceeded a social-ecological system crosses a threshold—resulting in an alternate natural resource regime that may be less desirable than the previous one. Flexibility within multi-level and cross-sectoral coordination mechanisms are necessary to allow for some degree of adaptation by decision makers to changes in social-ecological systems, or to allow for transformation if conditions have degraded to an undesirable condition (Jozaei et al. 2022).

We have identified the potential in existing laws and regulations for multi-level and cross-sectoral coordination of natural resource governance, and demonstrated how to identify existing, untapped legal capacity to promote coordinated natural resource governance. However, the question then arises as to how these untapped capacities within existing legal structures may be operationalized. Indeed, though these legal mechanisms are present, researchers in the fields of ecology, governance, and sustainability sciences continue to find an absence of coordination in practice. We propose three possible reasons for this discrepancy between law and practice: (1) lack of enforcement of the coordination obligation, (2) the coordination mechanism does not work in practice, or (3) the coordination mechanism does work in practice but is only useful in particular circumstances. Each possibility requires brief elaboration.

Lack of enforcement, at least in the context of our present case study, is not a result of an absence of enforcement mechanisms within the law. As demonstrated above, given that the law has clear obligations to coordinate in a number of circumstances, failure to do so may invalidate the policy made or public decision taken for contravening the applicable law. Even where the enabling law does not include a direct provision requiring coordination, the failure to do so by government departments such that conflicting policy or decision-making results may be subjected to constitutional review and invalidated on the basis of failure to comply with the Constitution’s section 41 obligation to cooperate and coordinate. In South Africa, there is untapped capacity for coordination in existing law given that enforcement is possible. Tapping into this capacity requires cases to be taken to court and will only be pursued by a party where it suits their own interests to do so, and so arises an issue of lack of enforcement in South Africa.

It could alternatively be that the coordination mechanisms in the legal framework do not actually work: consultation and coordination does not necessarily promote shared understanding. It does not even guarantee the results of the consultation and coordination are taken into account in decision-making. If so, we need more guidance on what type of mechanism(s) would be useful in promoting coordination, beyond the very global guidance prevalent in the existing state of the art as summarized in Table 2. This is thus not a case of the existing regulatory regime failing to enable coordination, but rather an issue with the type and quality of such coordination that represents an important topic for future empirical research.

Finally, it could be that the coordination mechanisms, in this case those identified in South African law, do actually promote coordination, but it is dependent on specific circumstances. Given that public authorities can (and have to) pick and choose, they can make errors and select a less appropriate coordination mechanism in practice. If this is the case, the untapped capacity within the legal system may be tapped once guidance on how to best use existing mechanisms is researched and compiled.

## Conclusion

When considering the legal framework for water resource governance as positioned within the broader natural resource governance regime in South Africa, there clearly exists potential for promoting coordination across sectors and levels of government. The potential for coordination derives from identifying untapped capacities in the laws and regulations (Garmestani et al. 2019), and our analysis reveals these untapped capacities to promote coordination of water resource governance across a number of legal and policy mechanisms.

The protocol developed in this paper to assess laws for untapped capacity to coordinate decision-making across sectors and levels of governance within the natural resource governance regime (as demonstrated through a case study of the water resource governance regime) is novel and agenda-setting. Our analysis makes clear that legal reform is, at least in South African law, not essential given that there is untapped formal capacity in existing laws to promote coordinated governance of water resources. The research presented here was focused on South Africa, but the protocol developed for this manuscript is portable to other countries around the world and to other environmental problems, further demonstrating its utility for research on formal aspects of environmental governance. This is a pathbreaking discovery and insight, and our analysis has the potential to be used throughout the world to assess legal frameworks for capacity to promote coordinated natural resource governance.

## Figures and Tables

**Table 1:** List of documents included in document review

Overarching legislation and policy	Status
1. Constitution of the Republic of South Africa	Supreme Law
*National level*	
2. Promotion of Administrative Justice Act 3 of 2000	Primary
3. National Development Plan 2030	Policy
4. Disaster Management Act 57 of 2002	Primary
5. National Environmental Management Act 107 of 1998 (as amended)	Primary
6. Intergovernmental Fiscal Relations Act 97 of 1997	Primary
7. Intergovernmental Relations Framework Act 13 of 2005	Primary
8. Local Government: Municipal Systems Act 32 of 2000	Primary
*Provincial level*	
9. Western Cape Provincial Disaster Management Framework—October 2007	Subordinate
10. Western Cape Policy on Public Participation—October 2010	Policy
*Municipal level*	
11. Disaster Risk Management Plan (City of Cape Town Municipality)	Policy
Sectoral Legislation and Policy	
*National Level*	
12. Water Services Act 108 of 1997	Primary
13. Water Services Amendment Act 30 of 2004	Primary
14. Regulations Relating to Compulsory National Standards and Measures to Conserve Water	Subordinate
15. Regulations on Norms and Standards in Respect of Tariffs for Water Services	Subordinate
16. Guidelines for Norms and Standards for Water Services Tariffs *(non-binding, interpretative aid for the regulations)*	Guidelines
17. Water Services Provider Contract Regulations (No. R. 980 of 2002)	Subordinate
18. Strategic Framework for Water Services *(non-binding—comprehensive summary of policy regarding water services)*	Guidelines
19. National Water Act 36 of 1998	Primary
20. National Water Amendment Act (No. 45 of 1999)	Primary
21. National Water Resource Strategy 2004 (BINDING)	Subordinate
22. National Water Resource Strategy 2013 (BINDING)	Subordinate
23. Regulations on the Use of Water for Mining and Related Activities Aimed at the Protection of Water Resources (Gazette No. 32935—Regulation 77)	Subordinate
24. Notice No. 131 of 2017 Regulations requiring that the taking of water for irrigation purposes be measured, recorded and reported	Subordinate
25. Establishment of a Pricing Strategy for Water Use Charges [i.t.o. s.56(1) NWA] 1998 (R. 1351)	Subordinate
26. Regulations on Use of Water for Mining and Related Activities Aimed at the Protection of Water Resources (No. 704)	Subordinate
27. Revision of General Authorisations [i.t.o s.39 NWA] 1998 (Notice No. 399 of 2004)	Subordinate
28. Replacement of General Authorisations [i.t.o s.39 NWA] 1998 (GN No. 1199 of 2009)	Subordinate
29. General Authorisations in terms of section 39 of the National Water Act, 1998 (Notice No. 398 of 2004)	Subordinate
30. General Authorisations in Terms of section 39 of the National Water Act, 1998 (No. 1191)	Subordinate
31. Regulations on financial assistance to resource poor farmers (No. R. 1036 of 2007)—(in support of agricultural water use)	Subordinate
32. Water Tribunal Rules (Notice No. 926 of 2005)	Subordinate
33. Water Use Registration Regulations (R. 1352) 1999	Subordinate
34. Revision of General Authorisation in terms of section 39 of the National Water Act, 1998 (GN No. 665 of 2013)	Subordinate
35. Water Use License Application and Appeals Regulations, 2017 (No. R. 267 of 2017)	Subordinate
36. Revision of general authorisation for taking and storing of water (Notice No. 538 of 2016)	Subordinate
37. The Water Research Act 34 of 1971	Primary
*Local level*	
38. Recreational Water Use By-law (2018)	Subordinate
39. Water By-law—October 2010	Subordinate
40. Limit or Restrict the Use of Water By-law—February 2003	Subordinate
41. Water Amendment By-law, 2018	Subordinate
42. Environmental Health By-law 2011	Subordinate
43. Stormwater Management By-law—August 2005	Subordinate
44. Cape Town Water Strategy—January 2019	Strategy
45. The Water Services Development Plan 2010/11—2013/14	Policy
46. Water and Sanitation Departmental Business Plan 2019/20	Policy
47. Water Conservation and Demand Management Strategy—March 2015	Strategy

**Table 2:** Protocol for identifying legal and/or policy mechanisms with capacity to promote coordination of governance

Characteristic of a regulatory framework with capacity to promote interorganizational coordination	Literature identifying characteristic
Information sharing across governmental departments and across levels of government	Allen et al. (2014), Bhaduri et al. (2015), Cosens and Gunderson (2018), Daher and Mohtar (2015), Endo et al. (2017), Feindt and Weiland (2018), Garmestani et al. (2009), Gregory et al. (2006), Harvey (2023), Kaddoura and El Khatib (2017), Liu et al. (2017), Ruhl (2012), Scott et al. (2015), Simpson and Jewitt (2019)
Multi-stakeholder engagement in policy, law-making and/or decision-making process	Bhaduri et al. (2015), Gottschick (2018), Gregory et al. (2006), Laspidou et al. (2020), McNutt and Rayner (2018), Mohtar and Daher (2016), Olawuyi (2020), van Gevelt (2020), White et al. (2017)
Inter-sectoral policy making	Daher and Mohtar (2019), Durnova (2018), Gregory et al. (2006), Howarth and Monasterolo (2016), Liu et al. (2018), White et al. (2017)
Bridging and/or cross-cutting mechanisms: e.g., establishment of formal spaces for coordination, or procedures incorporating coordination practices	Allen et al. (2014), Cosens and Gunderson (2018), Durnova (2018), Garmestani et al. (2009), Gilissen et al. (2016), Gottschick (2018), McNutt and Rayner (2018), Ruhl (2012)
Harmonization or coherence in sectoral goals and/or objectives	Covarrubias (2019), van Gevelt (2020)
Consistent monitoring (of harmonized goals and/or objectives)	Bandala and Berli (2018), Beijen et al. (2014), Biggs et al. (2015), Brethaut et al. (2019), Holling (1973)
Consideration of inter-sectoral issues in legislation and planning	Daher and Mohtar (2019), Howarth and Monasterolo (2016), Liu et al. (2018), White et al. (2017)
Incentives for coordination in decision-making processes	D’Odorico et al. (2018), Kurian (2017), Laspidou et al. (2020), Villamayor-Tomas et al. (2015)
Sufficient budgetary planning for coordination in decision-making processes	Daher et al. (2019), Harvey (2023), Walters (2007)

**Table 3:** Results of doctrinal analysis of South African law and policy reviewed for capacity to promote coordination of governance

Characteristic of a regulatory framework with capacity to promote interorganizational coordination	Number of documents with 1 or more provisions reflecting the characteristics
Information sharing across governmental departments and across levels of government	12
Multi-stakeholder engagement in policy and law-making process	10
Inter-sectoral policy making	7
Bridging and/or cross-cutting mechanisms: e.g., establishment of formal spaces for coordination, or procedures incorporating coordination practices	6
Harmonization or coherence in sectoral goals and/or objectives	2
Consistent monitoring (as a means of ensuring compliance with the various obligations to coordinate)	23
Consideration of inter-sectoral issues in legislation and planning	22
Incentives for coordination in decision-making processes	13
Sufficient budgetary planning for coordination in decision-making processes	7

**Table 4:** Categories of mechanisms with capacity to promote coordination in the overarching legislative and policy framework

Category	Coordination characteristic(s)	Example from document review
1. Establishment of a coordinating body	Bridging mechanism Information sharing Multi-stakeholder engagement	The establishment of the Committee for Environmental Coordination (section 7(1) National Environmental Management Act) comprised of Director Generals representing multiple resource sectors including the Director Generals of Water Affairs and Forestry, of Energy,and of Agriculture
		The establishment of the President’s Coordinating Council (“Conclusion” and 7 Intergovernmental Relations Framework Act) in which matters of national interest are raised and discussed with provincial governments and organized municipal government and in which the national government consults with provincial and municipal government on the implementation and coordination of policy and legislation
2. Mandates for cross-scale and/or cross-sectoral consultations and/or considerations embedded within procedural rules for legislation and/or policy formulation	Information sharing Multi-stakeholder engagement Consideration of inter-sectoral issues	Section 24(1) of the Municipal Systems Act requires that planning undertaken by a municipality be aligned with and complement ‘the development plans and strategies of other affected municipalities and other organs of state to give effect to the principles of co-operative government contained in section 41 of the Constitution’
		Section 1.1.4 of the Western Cape Provincial Disaster Management Framework expressly mandates inter-sectoral coordination through the requirement that the policy made at provincial level relating to disaster management be submitted to the relevant national sectoral cluster committees for ‘assessment and recommendations’ given the ‘multisectoral nature of disaster risk management’
3. Oversight and monitoring obligations embedded in procedural rules	Consistent monitoring	“Results”(3)(b) of the Disaster Management Act provides that the Intergovernmental Committee on Disaster Management is accountable to the national Cabinet and must ‘report to Cabinet on the co-ordination of disaster management among the spheres of government
		Section 16(2)(a) of the National Environmental Management Act grants power on the Director -General to monitor compliance with environmental implementation plans and environmental management plans and may take any steps or make any inquiries he or she deems fit to determine if environmental implementation plans and environmental management plans are being complied with by organs of state
4. Empowerment of organs of state to enter into, and set the parameters of, implementation protocols	Bridging mechanism	Section 35 of the Intergovernmental Relations Framework Act enables organs of state to enter into an implementation protocol that defines ‘the roles and responsibilities of each organ of state in implementing policy, exercising the statutory power, performing the statutory function or providing the [public] service’
5. Procedural requirements for public participation	Information sharing Multi-stakeholder engagement	Overarching procedural frameworks can be found in “Discussion”(l)(a), 16(1) and 17–22 in the Municipal Systems Act for participation in policy and decision-making at municipal level, and in the Western Cape Policy on Public Participation in policy making at provincial level
		Designated procedures in specific legislation for public participation in legislative and policy making and in decision-making processes such as the section 7(2)(f) requirement of the Disaster Management Act that disaster management frameworks facilitate public participation and multi-stakeholder engagement in disaster management
6. Express requirement that coordination take place in the development of policy and planning documents	Information sharing Multi-stakeholder engagement Harmonization or coherence in sectoral goals and/or objectives	“Background and context”(4)(l) of the National Environmental Management Act mandates ‘intergovernmental co-ordination and harmonization of policies, legislation and actions relating to the environment’
		The National Environmental Management Act further requires departments conducting functions that impact the natural environment (as defined in the Schedules to the Act and which includes the Department of Water Affairs) to draft environmental implementation plans or environmental management plans, as the case may be. Per section 12 of the Act, the plans are utilized to ‘co-ordinate and harmonize the environmental policies, plans, programmed and decisions of the various national departments’ through the requirement that in developing these plans, governmental departments ‘take into consideration every other [EIP] and [EMP] already adopted with a view to achieving consistency among such plans
7. Guiding principles	Harmonization or coherence in sectoral goals and/or objectives Inter-sectoral policy making	Section 195 of the Constitution sets out the basic values and principles governing public administration which includes requirements for public administration to promote the ‘[e]fficient, economic and effective use of resources’ (section 195(1) (b)) and the impartial, fair, equitable, and unbiased provision of services (section 195(1)(d))
		“Background and context” of the National Environmental Management Act provides for cooperative natural resource governance by establishing principles for decision-making on matters affecting the environment premised upon the underlying aim of the Act to coordinate environmental functions exercised by organs of state
8. Legal obligations for adequate budgetary planning	Sufficient budgetary planning	Present in every document reviewed
		Intergovernmental Fiscal Relations Act is the core document in this regard. Establishes a Budget Council (“Background and context”) in which the national government and the provincial governments consult on any fiscal, budgetary or financial matter affecting the provincial sphere of government including financial implications of legislation and policy and financial monitoring (“Methods”). Further establishes Local Government Budget Forum (“Discussion”) is a body in which the national government, the provincial governments and organized local government consult on any fiscal, budgetary or financial matter affecting the provincial sphere of government including financial implications of legislation and policy and financial monitoring (“Conclusion”)
9. Discretionary powers	Information sharing Multi-stakeholder engagement Consideration of inter-sectoral issues in legislation and planning	The Disaster Management Act establishes advisory forums at national (“Discussion”(3)), provincial (section 37), and municipal (section 51) levels which serves as a forum for government and stakeholders to ‘consult one another and co-ordinate their actions on matters relating to disaster management’ (“Discussion”(3)). The Act requires that a ‘wide range’ of stakeholders are included, but grants the Minister of Cooperative Government the discretionary power to determine precisely which stakeholders and government sectors to include
		Section 9(1) of the Intergovernmental Relations Framework Act states that ‘Any Cabinet member may establish a national intergovernmental forum to promote and facilitate intergovernmental relations in the functional area for which that Cabinet member is responsible’
10. Performance management indicators	Consistent monitoring Incentives for coordination	Section 49 of the Municipal Systems Act 32 of 2000 mandates establishment of performance management indicators and systems and prescribes a number of monitoring requirements thereof

**Table 5:** Categories of mechanisms with capacity to promote coordination in the sectoral legislative and policy framework

Category	Coordination characteristic(s)	Example from document review
1. Establishment of a coordinating body	Bridging mechanism Information sharing Multi-stakeholder engagement	Section 3.6.4 Strategic Framework for Water Services: ‘The Department of Provincial and Local Government (DLPG) has overall responsibility for the affairs of local government. This includes policy, legislation, capacity building, grant allocation and regulation as these apply to the integrated aspects of municipal services provision, including governance, administration, municipal finance, and integrated planning. Many of these responsibilities are exerted through provincial government.’ [DLPG’s overarching coordinating power which includes limited regulatory power and fund allocation, and management represents a potential body to promote more inter-sectoral policy making and decision-making]
		Chapter 7 of the National Water Act empowers the Minister to establish catchment management agencies with the purpose of ‘delegating water resource management to the regional or catchment level and to involve local communities’; the function of the agency per section 80 includes to ‘co-ordinate the related activities of water users and of the water management institutions within its water management area; [and] promote the co-ordination of its implementation with the implementation of any applicable development plan established in terms of the Water Services Act’
2. Mandates for cross-scale and/or cross-sectoral consultations and/or considerations embedded within procedural rules for legislation and/or policy formulation	Information sharing Multi-stakeholder engagement Consideration of inter-sectoral issues	Section 138 National Water Act: ‘The Minister must, after consultation with relevant- (a) organs of state; (b) water management institutions; and (c) existing and potential users of water, establish mechanisms and procedures to co-ordinate the monitoring of water resources’
		Explanatory Note p1 of Regulations on the Use of Water for Mining and Related Activities Aimed at the Protection of Water Resources: ‘The Department [of Water Affairs and Forestry] subscribes to the principle of co-operative governance and recognizes the role of the Department of Minerals and Energy to co-ordinate environmental management within the mining industry and the role of the Department of Environmental Affairs and Tourism as the lead agent of matters affecting the environment. … To promote coordination, copies of the relevant exemptions from the requirements of the regulations will be forwarded to the Department of Minerals and Energy and the Department of Environmental Affairs and Tourism.’
3. Oversight and monitoring obligations embedded in procedural rules	Consistent monitoring	Section 62(1) Water Services Act: ‘The Minister and any relevant Province must monitor the performance of every water services institution in order to ensure’ compliance with all applicable standards detailed in (a)–(c) of the section. Section 67 then establishes a national information system the purpose of which is set out in section 68 as being: ‘to record and provide data for the development, implementation and monitoring of national policy on water services…’
		The entire section 8 of the Strategic Framework for Water Services, and particularly section 8.4, is dedicated to developing a monitoring strategy; for example: ‘The monitoring system for the purposes of evaluating overall sector progress should be designed around the sector vision, goals and targets set out in “Background and context” of this Strategic Framework.’
4. Implementation protocols		Not present in sectoral legislation
5. Procedural requirements for public participation	Information sharing Multi-stakeholder engagement	Section 80 National Water Act: ‘Subject to Chapter 2 and section 79, upon establishment of a catchment management agency, the initial functions of a catchment management agency are- … (e) to promote community participation in the projection, use development, conservation, management and control of water resources in its water management area’
		Section 4.3.1 National Water Resource Strategy 2004: ‘The Department will continue to undertake public consultation exercises in a way that enables all stakeholders, particularly those from previously disadvantaged backgrounds, to participate effectively. Help will be offered to ensure that they understand the issues under discussion and can participate in an informed and meaningful way’
6. Express requirement that coordination take place in the development of policy and planning documents	Information sharing Multi-stakeholder engagement Harmonization or coherence in sectoral goals and/or objectives	Section 10(2) National Water Act: ‘In developing a catchment management strategy, a catchment management agency must consult with…(b) any organ of State which has an interest in the content, effect or implementation of the catchment management strategy. ‘
		Section 22(4) National Water Act: ‘In the interests of co-operative governance, a responsible authority may promote arrangements with other organs of state to combine their respective license requirements into a single license requirement.’
		Not strictly a legal obligation given it is within the Strategic Framework for Water Services. However, since this framework is considered a ‘comprehensive summary of prevailing policy’ it carries much weight in interpreting and guiding the development of law and policy in the water sector. Section 1.4 states that ‘[i]t is important that there be alignment between policies, legislation and strategies within the water services sector as well as alignment between these and the policies, legislation and strategies of other sectors related to the water sector (for example, water resources, finance, local government, housing and health)’
7. Guiding principles		Not present in sectoral legislation
8. Legal obligations for adequate budgetary planning	Sufficient budgetary planning	Section 7 of Pricing Strategy for Water Use Charges requires adequate budgetary planning to maintain water infrastructure: ‘Water resource development and use of waterworks refer to the planning, design, construction, operation, maintenance, refurbishment and betterment (improvement) of Government water schemes and schemes to be funded by water management …. If water use charges are too low, they will lead to underinvestment, over-consumption and unwarranted fiscal subsidies. There is therefore a need to adjust to higher real tariffs over time to accommodate the cost of investing in supply capacity to meet rising demand and to refurbish existing infrastructure.’
9. Discretionary powers	Information sharing Multi-stakeholder engagement Consideration of inter-sectoral issues in legislation and planning	Section 10(1) Water Services Act: ‘The Minister may, with the concurrence of the Minister of Finance, from time to time prescribe norms and standards in respect of tariffs for water services. Section 10(2)(d): ‘These norms and standards may provide for tariffs to be used to promote or achieve water conservation.’
		Section 67 National Water Act grants the Minister power, ‘in an emergency situation or in cases of extreme urgency involving the safety of humans or property or the protection of a water resource or the environment’ to dispense with public comment prior to publication, notice periods and time limits and authorize a water management institution to dispense of these requirements as well
10. Performance management indicators	Consistent monitoring Incentives for coordination	“Discussion”(l) Water Services Provider Contract Regulations: ‘A contract [with Water Service Provider] must provide for—(a) performance targets and indicators developed after consultation with consumers, including those relating to the levels of service and standards of service to be achieved by the water services provider over fixed periods’
		The non-binding guidelines which serve as an interpretive aid for the Regulations Relating to Compulsory National Standards and Measures to Conserve Water explain that the section 10 obligation that the water services authority must include a water services audit in its annual report enables the use of the audit as a tool to compare actual performance of the water services authority against the targets and indicators set in their plan. The water audit can also be used to determine opportunities, the role and key performance indicators of water conservation and water demand management
11. Framework for governing a particular inter-sectoral issue	Inter-sectoral policy making Consideration of cross-sectoral issues	The Regulations on Use of Water for Mining and Related Activities Aimed at the Protection of Water Resources (No. 704) provides a clear framework for water use and conservation within the mining sector, while the Regulations on Financial Assistance to Resource Poor Farmers (No. R. 1036 of 2007) establishes a framework for providing subsidized water to support agricultural water use by disadvantaged farmers

## Footnotes

1 Available at https://www.gov.za/document/latest.

2 Available at https://www.capetown.gov.za/Docum ent-centre.

## References

[R1] Akamani K (2016) Adaptive water governance: integrating the human dimensions into water resource governance. J Contemp Water Res Educ 158:2–18. 10.1111/j.1936-704X.2016.03215.x

[R2] Allen CR, Gunderson LH (2011) Pathology and failure in the design and implementation of adaptive management. J Environ Manag Adapt Manag Nat Resour 92:1379–1384. 10.1016/j.jenvman.2010.10.063 21112687

[R3] Allen CR, Fontaine JJ, Pope KL, Garmestani AS (2011) Adaptive management for a turbulent future. J Environ Manag Adapt Manag Nat Resour 92:1339–1345. 10.1016/j.jenvman.2010.11.019 21168260

[R4] Allen CR, Angeler DG, Garmestani AS, Gunderson LH, Holling CS (2014) Panarchy: theory and application. Ecosystems 17:578–589. 10.1007/s10021-013-9744-2

[R5] Bandala ER, Berli M (2018) Engineered nanomaterials (ENMs) and their role at the nexus of Food, Energy, and Water. Mater Sci Energy Technol 2:29–40. 10.1016/j.mset.2018.09.004

[R6] Beijen BA, van Rijswick HFMW, Anker HT (2014) The importance of monitoring for the effectiveness of environmental directives: a comparison of monitoring obligations in European environmental directives. Utrecht Law Rev 10:126

[R7] Bergendahl JA, Sarkis J, Timko M (2018) Transdisciplinarity and the food energy and water nexus: ecological modernization and supply chain sustainability perspectives. Resour Conserv Recycl 133:309–319

[R8] Bhaduri A, Ringler C, Dombrowski I, Mohtar R, Scheumann W (2015) Sustainability in the water–energy–food nexus. Water Int 40:723–732. 10.1080/02508060.2015.1096110

[R9] Biggs EM, Bruce E, Boruff B, Duncan JMA, Horsley J, Pauli N, McNeill K, Neef A, Ogtrop FV, Curnow J, Haworth B, Duce S, Imanari Y (2015) Sustainable development and the water-energy-food nexus: a perspective on livelihoods. Environ Sci Policy 54:389–397. 10.1016/j.envsci.2015.08.002

[R10] Boström M, Grönholm S, Hassler B (2016) The ecosystem approach to management in Baltic Sea governance: towards increased reflexivity? In: Gilek M, Karlsson M, Linke S, Smolarz K (eds) Environmental governance of the Baltic Sea, MARE Publication Series. Springer International Publishing, Cham, pp 149–172. 10.1007/978-3-319-27006-7

[R11] Bouckaert G, Peters G, Verhoest K (2010) The coordination of public sector organizations, 1st edn. Palgrave Macmillan, London

[R12] Bréthaut C, Gallagher L, Dalton J, Allouche J (2019) Power dynamics and integration in the water-energy-food nexus: learning lessons for transdisciplinary research in Cambodia. Environ Sci Policy 94:153–162. 10.1016/j.envsci.2019.01.010

[R13] Chaffin BC, Garmestani AS, Gunderson LH, Benson MH, Angeler DG, Arnold CA (Tony), Cosens B, Craig RK, Ruhl JB, Allen CR (2016) Transformative environmental governance. Annu Rev Environ Resour 41(1):399–423. 10.1146/annurev-environ-110615-085817 32607083 PMC7326237

[R14] Clement S (2022) Knowledge governance for the Anthropocene: pluralism, populism, and decision-making. Glob Policy 13(S3):11–23. 10.1111/1758-5899.13148

[R15] Cosens B, Gunderson L (eds) (2018) Practical panarchy for adaptive water governance. Springer International Publishing, Cham. 10.1007/978-3-319-72472-0

[R16] Cosens BA, Craig RK, Hirsch SL, Arnold CA, Benson MH, DeCaro DA, Garmestani AS, Gosnell H, Ruhl JB, Schlager E (2017) The role of law in adaptive governance. Ecol Soc 22:1–30. 10.5751/ES-08731-220130 PMC5954422 29780426

[R17] Covarrubias M (2019) The nexus between water, energy and food in cities: towards conceptualizing socio-material interconnections. Sustain Sci 14:277–287. 10.1007/s11625-018-0591-0

[R18] Craig RK (2010) “Stationarity Is Dead”—long live transformation: five principles for climate adaptation law. Harv Environ Law Rev 34(1):9–73

[R19] Craig RK, Ruhl JB, Garmestani A (2020) The flexibility of existing laws is an essential element of environmental governance. Proc Natl Acad Sci 117:8245–8246 32234792 10.1073/pnas.1922201117 PMC7165482

[R20] D’Odorico P, Davis KF, Rosa L, Carr JA, Chiarelli D, Dell’Angelo J, Gephart J, MacDonald GK, Seekell DA, Suweis S, Rulli MC (2018) The global food-energy-water nexus. Rev Geophys 56:456–531. 10.1029/2017RG000591

[R21] Daher BT, Mohtar RH (2015) Water–energy–food (WEF) Nexus Tool 2.0: guiding integrative resource planning and decision-making. Water Int 40:748–771. 10.1080/02508060.2015.1074148

[R22] Daher B, Mohtar RH (2019) Toward creating an environment of cooperation between water, energy, and food stakeholders in San Antonio. Sci Total Environ 651:2913–2926. 10.1016/j.scitotenv.2018.09.395 30463143

[R23] Daher B, Lee S-H, Blake J, Shafiezadeh H, Zamaripa S, Mohtar RH (2019) Towards bridging the water gap in Texas: a water-energy-food nexus approach. Sci Total Environ 647:449–463. 10.1016/j.scitotenv.2018.07.398 30086497

[R24] Dedeurwaerdere T (2005) From bioprospecting to reflexive governance. Ecol Econ Biodivers Conserv Access Benefit Shar Tradit Knowl 53:473–491. 10.1016/j.ecolecon.2004.10.013

[R25] Dryzek JS, Pickering J (2017) Deliberation as a catalyst for reflexive environmental governance. Ecol Econ 131:353–360. 10.1016/j.ecolecon.2016.09.011

[R26] Dubow S (2014) The Personal is Political—Apartheid, 1948–1994. Oxford University Press

[R27] Durnova A (2018) A tale of ‘fat cats’ and ‘stupid activists’: contested values, governance and reflexivity in the brno railway station controversy. J Environ Plan Policy Manag 20:720–733. 10.1080/1523908X.2013.829749

[R28] Dworkin R (1986) Law’s empire. Belknap Press, Cambridge

[R29] Endo A, Oh T (eds) (2018) The water-energy-food nexus: human-environmental security in the Asia-Pacific ring of fire, 1st edn. Springer, Singapore

[R30] Endo A, Tsurita I, Burnett K, Orencio PM (2017) A review of the current state of research on the water, energy, and food nexus. J Hydrol Reg Stud 11:20–30. 10.1016/j.ejrh.2015.11.010

[R31] Enqvist J, Ziervogel G, Metelerkamp L, van Breda J, Dondi N, Lusithi T, Mdunyelwa A, Mgwigwi Z, Mhlalisi M, Myeza S, Nomela G, October A, Rangana W, Yalabi M (2020) Informality and water justice: community perspectives on water issues in Cape Town’s low-income neighbourhoods. Int J Water Resour Dev 38(1):108–129. 10.1080/07900627.2020.1841605

[R32] Feindt P (2012) Reflexive governance, public goods and sustainability—conceptual reflections and empirical evidence in agricultural policy. In: Brousseau E, Dedeurwaerdere T, Siebenhuner B (eds) Reflexive governance for global public goods. MIT Press, Cambridge, pp 159–178

[R33] Feindt PH, Weiland S (2018) Reflexive governance: exploring the concept and assessing its critical potential for sustainable development. Introduction to the special issue. J Environ Plan Policy Manag 20:661–674. 10.1080/1523908X.2018.1532562

[R34] Folke C, Hahn T, Olsson P, Norberg J (2005) Adaptive governance of social-ecological systems. Annu Rev Environ Resour 30:441–473. 10.1146/annurev.energy.30.050504.144511

[R35] Francis D, Webster E (2019) Inequality in South Africa. Dev South Afr 36:733–734. 10.1080/0376835X.2019.1699397

[R36] Gamper A (2010) On loyalty and the (federal) constitution. ICL J 4:157–170. 10.1515/icl-2010-0203

[R37] Garmestani A, Benson M (2013) A framework for resilience-based governance of social-ecological systems. Ecol Soc. 10.5751/ES-05180-180109

[R38] Garmestani AS, Allen CR, Cabezas H (2009) Panarchy, adaptive management and governance: policy options for building resilience. Nebraska Law Rev 87:20

[R39] Garmestani A, Craig RK, Gilissen HK, McDonald J, Soininen N, van Doorn-Hoekveld WJ, van Rijswick HFMW (2019) The role of social-ecological resilience in coastal zone management: a comparative law approach to three coastal nations. Ecol Evol Front. 10.3389/fevo.2019.00410 PMC7970458 33748149

[R40] Gilissen HK, Alexander M, Beyers J-C, Chmielewski P, Matczak P, Schellenberger T, Suykens C (2016) Bridges over troubled waters: an interdisciplinary framework for evaluating the interconnectedness within fragmented flood risk management systems. J Water Law 25:12–26

[R41] Gottschick M (2018) Reflexive capacity in local networks for sustainable development: integrating conflict and understanding into a multi-level perspective transition Framework. J Environ Plan Policy Manag 20:704–719. 10.1080/1523908X.2013.842890

[R42] Gregory R, Ohlson D, Arvai J (2006) Deconstructing adaptive management: criteria for applications to environmental management. Ecol Appl 16:2411–2425. 10.1890/1051-0761(2006)016[2411:DAMCFA]2.0.CO;2 17205914

[R43] Gulati R, Wohlgezogen F, Zhelyazkov P (2012) The two facets of collaboration: cooperation and coordination in strategic alliances. ANNALS 6:531–583. 10.5465/19416520.2012.691646

[R44] Gunderson LH, Holling CS (2002) Panarchy: understanding transformations in human and natural systems. Island Press, Washington

[R45] Harvey N (2023) Operationalizing the water, energy and food nexus through the law. In: Duque de Brito PS, da Costa Sanches Galvão JR, Monteiro P, Panizio R, Calado L, Assis AC, dos Santos Neves F, Craveiro F, de Amorim Almeida H, Correia Vasco JO, de Jesus Gomes R, de Jesus Martins Mourato S, Santos Ribeiro VS (eds) Proceedings of the 2nd international conference on water energy food and sustainability (ICoWEFS 2022). Springer International Publishing, Cham, pp. 451–469. 10.1007/978-3-031-26849-6_47

[R46] Hoecke MV (2011) Legal doctrine: which method(s) for what kind of discipline? In: Hoecke MV (ed) Methodologies of legal research: what kind of method for what kind of discipline? Hart Publishing, London, pp 1–18

[R47] Hoff H (2011) Understanding the Nexus. Background paper for the Bonn2011 Nexus Conference. Presented at the Bonn 2011 conference: the water energy and food security nexus—solutions for the Green Economy. Stockholm Environmental Institute, pp 51.

[R48] Holling CS (1973) Resilience and stability of ecological systems. Annu Rev Ecol Syst 4(1):1–23. 10.1146/annurev.es.04.110173.000245

[R49] Howarth C, Monasterolo I (2016) Understanding barriers to decision making in the UK energy–food–water nexus: the added value of interdisciplinary approaches. Environ Sci Policy 61:53–60

[R50] Huitema D, Mostert E, Egas W, Moellenkamp S, Pahl-Wostl C, Yalcin R (2009) Adaptive water governance: assessing the institutional prescriptions of adaptive (Co-)management from a governance perspective and defining a research agenda. Ecol Soc. 10.5751/ES-02827-140126

[R51] Hutchinson T (2015) The doctrinal method: incorporating interdisciplinary methods in reforming the law. Erasmus Law Rev. 10.5553/ELR.000055

[R52] Hutchinson T, Duncan N (2012) Defining and describing what we do: doctrinal legal research. Deakin Law Rev 17:83–119. 10.21153/dlr2012vol17no1art70

[R53] Jozaei J, Chuang W, Allen CR, Garmestani AS (2022) Social vulnerability, social-ecological resilience and coastal governance. Global Sustain 5:E12. 10.1017/sus.2022.10 PMC10304588 37383242

[R54] Kaddoura S, El Khatib S (2017) Review of water-energy-food nexus tools to improve the nexus modelling approach for integrated policy making. Environ Sci Policy 77:114–121. 10.1016/j.envsci.2017.07.007

[R55] Kirwan J, Maye D, Brunori G (2017) Reflexive governance, incorporating ethics and changing understandings of food chain performance. Sociol Rural 57:357–377. 10.1111/soru.12169

[R56] Kurian M (2017) The water-energy-food nexus: trade-offs, thresholds and transdisciplinary approaches to sustainable development. Environ Sci Policy 68:97–106. 10.1016/j.envsci.2016.11.006

[R57] Laspidou CS, Mellios NK, Spyropoulou A, Kofinas DT, Papadopoulou MP (2020) Systems thinking on the resource nexus: modeling and visualisation tools to identify critical interlinkages for resilient and sustainable societies and institutions. Sci Total Environ 717:137264 32092809 10.1016/j.scitotenv.2020.137264

[R58] Lawford R, Bogardi J, Marx S, Jain S, Wostl CP, Knüppe K, Ringler C, Lansigan F, Meza F (2013) Basin perspectives on the water–energy–food security nexus. Curr Opin Environ Sustain 5:607–616

[R59] Lemos MC, Agrawal A (2006) Environmental Governance. Annu Rev Environ Resour 31:297–325. 10.1146/annurev.energy.31.042605.135621

[R60] Lenschow A (2002) Environmental policy integration: greening sectoral policies in Europe. Routledge, London

[R61] Liu J, Yang H, Gosling S, Kummu M, Flörke M, Pfister S, Hanasaki N, Wada Y, Zhang X, Zheng C, Alcamo J, Oki T (2017) Water scarcity assessments in the past, present, and future: review on water scarcity assessment. Earth’s Future 5:545–559. 10.1002/eft2.2017.5.issue-6 30377623 PMC6204262

[R62] Liu J, Hull V, Godfray HCJ, Tilman D, Gleick P, Hoff H, Pahl-Wostl C, Xu Z, Chung MG, Sun J, Li S (2018) Nexus approaches to global sustainable development. Nat Sustain 1:466–476

[R63] Marsden T (2013) From post-productionism to reflexive governance: contested transitions in securing more sustainable food futures. J Rural Stud Food Secur 29:123–134. 10.1016/j.jrurstud.2011.10.001

[R64] McNutt K, Rayner J (2018) Is Learning without teaching possible? The productive tension between network governance and reflexivity. J Environ Plan Policy Manag 20:769–780. 10.1080/1523908X.2014.986568

[R65] Mees H, Alexander M, Gralepois M, Matczak P, Mees H (2018) Typologies of citizen co-production in flood risk governance. Environ Sci Policy 89:330–339. 10.1016/j.envsci.2018.08.011

[R66] Millington N, Scheba S (2021) Day zero and the infrastructures of climate change: water governance, inequality, and infrastructural politics in Cape Town’s water crisis. Int J Urban Reg Res 45(1):116–132. 10.1111/1468-2427.12899

[R67] Mohtar RH, Daher B (2016) Water international water-energy-food nexus framework for facilitating multi-stakeholder dialogue water-energy-food nexus framework for facilitating multi-stakeholder dialogue. Taylor Francis 41:655–661. 10.1080/02508060.2016.1149759

[R68] Olawuyi D (2020) Sustainable development and the water-energy-food nexus: legal challenges and emerging solutions. Environ Sci Policy 103:1–9. 10.1016/j.envsci.2019.10.009

[R69] Oldfield S, Greyling S (2015) Waiting for the state: a politics of housing in South Africa. Environ Planning A Econ Space 47(5):1100–1112. 10.1177/0308518X15592309

[R70] Peters BG (2017) What is so wicked about wicked problems? A conceptual analysis and a research program. Policy Soc 36:385–396. 10.1080/14494035.2017.1361633

[R71] Ruhl JB (2012) Panarchy and the law. Ecol Soc. 10.5751/ES-05109-170331

[R72] Ruhl JB, Fischman R (2010) Adaptive management in the courts. Minn Law Rev 437:424–484

[R73] Scarlett L (2013) Collaborative adaptive management: challenges and opportunities. Ecol Soc. 10.5751/ES-05762-180326

[R74] Scharpf FW (1994) Games real actors could play: positive and negative coordination in embedded negotiations. J Theor Polit 6:27–53. 10.1177/0951692894006001002

[R75] Schutter OD, Lenoble J (2010) Reflexive governance: redefining the public interest in a pluralistic world. Bloomsbury Publishing, London

[R76] Scott CA, Kurian M, Wescoat JL (2015) The water-energy-food nexus: enhancing adaptive capacity to complex global challenges. In: Kurian M, Ardakanian R (eds) Governing the nexus: water, soil and waste resources considering global change. Springer International Publishing, Cham, pp 15–38. 10.1007/978-3-319-05747-7_2

[R77] Serdeczny O, Adams S, Baarsch F, Coumou D, Robinson A, Hare W, Schaeffer M, Perrette M, Reinhardt J (2017) Climate change impacts in Sub-Saharan Africa: From physical changes to their social repercussions. Reg Environ Change 17(6):1585–1600. 10.1007/s10113-015-0910-2

[R78] Simpson GB, Jewitt GPW (2019) The development of the water-energy-food nexus as a framework for achieving resource security: a review. Environ Sci Front. 10.3389/fenvs.2019.00008

[R79] Sonnino R, Lozano Torres C, Schneider S (2014) Reflexive governance for food security: the example of school feeding in Brazil. J Rural Stud 36:1–12. 10.1016/j.jrurstud.2014.06.003

[R80] Sousa PM, Blamey RC, Reason CJC, Ramos AM, Trigo RM (2018) The `Day Zero’ Cape Town drought and the poleward migration of moisture corridors. Environ Res Lett 13(12):124025. 10.1088/1748-9326/aaebc7

[R81] Springer J, Campese J, Nakangu B (2021) The natural resource governance framework. IUCN. 10.2305/IUCN.CH.2021.16.en

[R82] Taekema S (2018) Theoretical and normative frameworks for legal research: putting theory into practice. Law Method. 10.5553/REM/.000031

[R83] van Gevelt T (2020) The water-energy-food nexus: bridging the science-policy divide. Curr Opin Environ Sci Health 13:6–10. 10.1016/j.coesh.2019.09.008

[R84] van Tatenhove JPM (2017) Transboundary marine spatial planning: a reflexive marine governance experiment? J Environ Plan Policy Manag 19:783–794. 10.1080/1523908X.2017.1292120

[R85] van Boom WH, Desmet P, Mascini P (2018) Empirical legal research: charting the terrain. In: van Boom WH, Desmet P, Mascini P (eds) Empirical legal research in action. Edward Elgar Publishing, London

[R86] Vatn A (2012) Environmental governance: the aspect of coordination. In: Brousseau E, Dedeurwaerdere T, Jouvet P-A, Willinger M (eds) Global environmental commons: analytical and political challenges in building governance mechanisms. OUP, Oxford

[R87] Villamayor-Tomas S, Grundmann P, Epstein G, Evans T, Kimmich C (2015) The water-energy-food security nexus through the lenses of the value chain and the institutional analysis and development frameworks. Water Altern 8:21

[R88] Voß J-P, Bornemann B (2011) The politics of reflexive governance: challenges for designing adaptive management and transition management. Ecol Soc. 10.5751/ES-04051-160209

[R89] Voß J-P, Bauknecht D, Kemp R (2006) Reflexive governance for sustainable development. Edward Elgar Publishing, London

[R90] Walker B, Holling CS, Carpenter S, Kinzig A (2004) Resilience, adaptability and transformability in social–ecological systems. Ecol Soc. 10.5751/ES-00650-090205

[R91] Walters CJ (1986) Adaptive management of renewable resources. Macmillan Publishers Ltd, Basingstoke

[R92] Walters C (1997) Challenges in adaptive management of riparian and coastal ecosystems. Conserv Ecol. 10.5751/ES-00026-010201

[R93] Walters CJ (2007) Is adaptive management helping to solve fisheries problems? AMBI 36:304–307. 10.1579/0044-7447(2007)36[304:IAMHTS]2.0.CO;2 17626467

[R94] Weitz N, Strambo C, Kemp-Benedict E, Nilsson M (2017) Closing the governance gaps in the water-energy-food nexus: Insights from integrative governance. Global Environ Change. 10.1016/j.gloenvcha.2017.06.006

[R95] Westling EL, Sharp L, Rychlewski M, Carrozza C (2014) Developing adaptive capacity through reflexivity: lessons from collaborative research with a UK water utility. Crit Policy Stud 8:427–446. 10.1080/19460171.2014.957334

[R96] White DD, Jones JL, Maciejewski R, Aggarwal R, Mascaro G (2017) Stakeholder analysis for the food-energy-water nexus in phoenix, Arizona: implications for nexus governance. Sustainability 9:2204. 10.3390/su9122204

[R97] Yin RK (2009) Case study research: design and methods. SAGE, Thousand Oaks

